# *Candida auris* screening, positivity trends, and patient characteristics at the University of Kentucky between 2021 and 2024

**DOI:** 10.1017/ash.2025.10151

**Published:** 2025-10-14

**Authors:** Faith Fursman, Natalie Fitzsimmons, DaNelle Overton, Court Desmond, Kimberly Blanton, Kevin W. Hatton, Rachel Howard, David Olafsson, Sean McTigue, Nicholas Van Sickels, Derek Forster, Takaaki Kobayashi

**Affiliations:** 1 Infection Prevention and Control, University of Kentucky Medical Center, Lexington, KY, USA; 2 College of Pharmacy, University of Kentucky, Lexington, KY, USA; 3 EVPHA Information Technology, University of Kentucky Medical Center, Lexington, KY, USA; 4 College of Medicine, University of Kentucky, Lexington, KY, USA; 5 Department of Anesthesiology, University of Kentucky Medical Center, Lexington, KY, USA

## Abstract

We evaluated a targeted *Candida auris* screening program, which revealed a 0.96% positivity rate. The proportion of community-onset cases increased from 8% to 54% following implementation. No significant differences in demographic or clinical characteristics were observed between patients with colonization and those with infection.

## Background

*Candida auris* is an emerging multidrug-resistant fungus recognized as a global public health threat.^1^ It has been implicated in multiple outbreaks worldwide, partly due to its high adaptability and intrinsic resistance to one or more classes of antifungal agents.^1,2^ Despite increasing colonization rates, the expansion of pilot screening programs, and high associated mortality, no standardized protocol exists in the United States for *C. auris* screening.^1,3– 5^ Notably, there is a paucity of studies evaluating the efficacy of *C. auris* screening in Southeastern or Appalachian states within the United States, highlighting a geographic gap in the current literature.^5–7^ Additionally. the absence of standardized screening guidelines presents challenges for infection prevention, timely intervention, and containment. In February 2023, the University of Kentucky Healthcare (UKHC) implemented a targeted *C. auris* screening program. This study aims to (A) identify risk factors and common characteristics associated with *C. auris* colonization and infection using data from the screening program and (B) assess temporal trends in positivity rates.

## Methods

This retrospective, observational study was conducted at UKHC, a 1,086-bed academic medical center, from July 1, 2021, to June 30, 2024. All adult patients (≥18 yr) tested for *C. auris* through either surveillance screening or clinical culture were included. The study was approved by the University of Kentucky Institutional Review Board. Before February 2023, *C. auris* screening was conducted exclusively during outbreak investigations. Following implementation, routine screening was expanded using axillary and groin swabs analyzed via polymerase chain reaction (PCR). Patients were screened if they met at least one of the following criteria: admission to an intensive care unit, admission from an outside facility with a wound or tracheostomy present on admission, or an active carbapenem-resistant organism infection. Surveillance screening was also performed in response to point prevalence testing when a patient was housed adjacent to an individual with newly identified *C. auris* who had not been under appropriate transmission-based precautions for more than 24 hours. Clinical cultures were defined as microbiological specimens, including respiratory, cerebrospinal fluid (CSF), blood, wound, or urine cultures, that yielded *C. auris*. For consistency in analysis, patients remained categorized according to their original classification, clinical or colonized, regardless of subsequent determinations by the care team. Cases were further classified as either community-onset, defined as sample collection before hospital day four, or hospital-onset, defined as sample collection on or after hospital day four, excluding emergency department visits. We compared patients with *C. auris* colonization to those with infection. χ^2^ and Fisher’s exact tests were used for categorical variables, and the Mann–Whitney U test was applied to continuous variables. We then conducted univariate logistic regression to identify factors associated with infection compared to colonization. Clinical variables found to be significant in univariate analysis were included in the multivariable analysis. A *P* value of <0.05 was considered statistically significant. The data analysis for this study was generated using SAS software, Version 9.4. Copyright © 2016, SAS Institute Inc.

## Results

**Figure 1.**
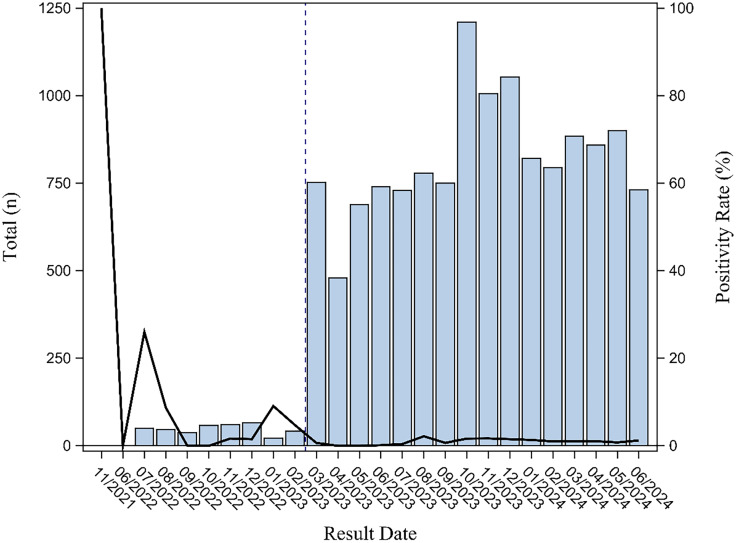
Testing Volume and Positivity Rate Before and After Implementing *Candida auris* Admission Screening at the University of Kentucky (2021–2024).

Between July 1, 2021, and June 30, 2024, 13,642 *C. auris* tests were performed, identifying 70 positive cases, including 13 preimplementation and 57 postimplementation (Figure 1). Among preimplementation cases, 7.69% were community-onset, whereas postimplementation community-onset cases accounted for 57.37% (Supplemental Table 1). Postimplementation monthly positivity rates ranged from 0% to 2.18%, with a mean of 0.96%. Among colonized patients, the primary reasons for testing were ICU admission (50.00%), point prevalence (20.00%), and wound (6.67%, Table 1). Among ten clinical cases, the most commonly identified clinical specimen types were bronchoalveolar lavage and urine (*n* = 5 each), with lower detection in invasive specimens (CSF = 1, blood = 2, Supplemental Table 2). Although *C. auris* was detected in clinical specimens, three cases did not receive anti-fungal treatment as the pathogen was deemed non-clinically significant. Thirty- and ninety-day mortality rates were comparable between clinical and colonized cases, with 30% versus 25% (*P* = 0.7377) and 40% versus 28.3% (*P* = 0.4561), respectively. No notable demographic differences were found between clinical and colonized patients. The mean age was 61.5 ± 15.2 years for clinical cases and 59.98 ± 14.7 years for colonized cases (*P* = 0.9557). Male sex predominated, accounting for 57.1% of the total cohort, with a higher, though statistically insignificant, proportion in clinical cases (80%) than in colonized cases (53.3%, *P* = 0.1147). The most common primary admission diagnoses were infectious diseases, neurology/neurosurgery, and pulmonary conditions, with no significant differences between clinical and colonized groups (*P* = 0.4035). In univariable analysis, significant risk factors for clinical cases included diabetes mellitus (*P* = 0.0143), osteomyelitis (*P* = 0.0273), tube feeding (*P* = 0.0293), tracheostomy or mechanical ventilation (*P* = 0.0404), and urinary diversion (*P* = 0.0121). These variables were included in the multivariable logistic regression model, in which none remained statistically significant.

**Table 1.** Demographics, past medical history, and admission and testing information for *Candida auris*positive patients

	Total Sample *n* = 70	Clinical *n* = 10	Colonization *n* = 60	*p*-value	MLR ^I^ Odds and CI	MLR ^I^ *p*-value
Age	60.20 (14.64)	61.50 (14.15)	59.98 (14.83)	0.9557		
Sex	0.1147					
Female	30 (42.86%)	2 (20%)	28 (46.67%)			
Male	40 (57.14%)	8 (80%)	32 (53.33%)			
Race	0.1163					
Black	8 (11.43%)	3 (30%)	5 (8.33%)			
Spanish American	3 (4.29%)	0 (0%)	3 (5.00%)			
White	59 (84.29%)	7 (70%)	52 (86.67%)			
Body-Mass Index (BMI)	31.05 (8.40)	34.96 (6.77)	30.40 (8.52)	0.4687		
Mortality Status						
30-Day	18 (25.71%)	3 (30%)	15 (25.00%)	0.7377		
3-Month	21 (30.00%)	4 (40%)	17 (28.33%)	0.4561		
Past Medical History						
Amputation	8 (11.43%)	1 (10%)	7 (11.67%)	0.8781		
Autoimmune	9 (12.86%)	1 (10%)	8 (13.33%)	0.7706		
Coronary Artery Disease	14 (20.00%)	4 (40%)	10 (16.67%)	0.0877		
Cirrhosis	8 (11.43%)	0 (0%)	8 (13.33%)	0.2198		
COPD ^a^ /Emphysema	26 (37.14%)	4 (40%)	22 (36.67%)	0.8399		
CRO ^b^	9 (12.86%)	3 (30%)	6 (10.00%)	0.0802		
Dementia	9 (12.86%)	2 (20%)	7 (11.67%)	0.4661		
Diabetes Mellitus	38 (54.29%)	9 (90%)	29 (48.33%)	0.0143	8.8 (0.9–86.4)	0.0622
End-Stage Renal Disease	5 (7.14%)	1 (10%)	4 (6.67%)	0.7047		
Heart Failure	21 (30.00%)	4 (40%)	17 (28.33%)	0.4561		
Hypertension	60 (85.71%)	9 (90%)	51 (85.00%)	0.6757		
Malignancy	9 (12.86%)	3 (30%)	6 (10.00%)	0.0802		
Osteomyelitis	15 (21.43%)	5 (50%)	11 (18.33%)	0.0273	1.47 (0.2–10.68)	0.7019
Stroke/Cerebrovascular Accident	16 (22.86%)	3 (30%)	13 (21.67%)	0.5612		
SUD/IVDU ^c^	25 (35.71%)	3 (30%)	22 (36.67%)	0.6838		
Wound	58 (82.86%)	9 (90%)	49 (81.67%)	0.5174		
Medical Devices						
Central Line	31 (44.29%)	6 (60%)	25 (41.67%)	0.2799		
Tracheotomy/Ventilation	35 (50.00%)	8 (80%)	27 (45.00%)	0.0404	3.42 (0.35–33.22)	0.289
Tube Feeding	41 (58.57%)	9 (90%)	32 (53.33%)	0.0293	4.24 (0.31–57.72)	0.278
Urinary Catheter	34 (48.57%)	6 (60%)	28 (46.67%)	0.4348		
Urinary Diversion	10 (14.29%)	4 (40%)	6 (10.00%)	0.0121	2.94 (0.28–30.49)	0.3655
Mobility Status	0.3265					
Ambulatory (limited) w/o Device	2 (2.86%)	0 (0%)	2 (3.33%)			
Device Use	15 (21.43%)	2 (20%)	13 (21.67%)			
Impaired Mobility/ADLs ^d^	9 (12.86%)	1 (10%)	8 (13.33%)			
No Impairment	27 (38.57%)	2 (20%)	25 (41.67%)			
Non-Ambulatory / Bed-Bound	17 (24.29%)	5 (50%)	12 (20.00%)			
Onset	0.0693					
Community	28 (40.00%)	2 (20%)	26 (43.33%)			
Hospital	33 (47.14%)	8 (80%)	25 (41.67%)			
Outside Hospital	9 (12.86%)	0 (0%)	9 (15.00%)			
Test Reason ^e^	<.0001					
Clinical Specimen	10 (14.29%)	10 (100%)	0 (0%)			
CRO ^b^ History	3 (4.29%)	0 (0%)	3 (5.00%)			
ICU ^f^ Admission	30 (42.86%)	0 (0%)	30 (50.00%)			
Point Prevalence	12 (17.14%)	0 (0%)	12 (20.00%)			
Wound	4 (5.72%)	0 (0%)	4 (6.67%)			
Admission Location	0.0030					
Acute Rehabilitation	2 (2.86%)	0 (0%)	2 (3.33%)			
Assisted Living	1 (1.43%)	0 (0%)	1 (1.67%)			
Home	21 (30.00%)	2 (20%)	19 (31.67%)			
Home w/ Home Health	2 (2.86%)	2 (20%)	0 (0%)			
Homeless	1 (1.43%)	0 (0%)	1 (1.67%)			
LTACH ^g^	1 (1.43%)	1 (10%)	0 (0%)			
Outside Hospital	30 (42.86%)	2 (20%)	28 (46.67%)			
SNF ^h^	12 (17.14%)	3 (30%)	9 (15.00%)			
Admission Service	0.4035					
Addiction Medicine	1 (1.43%)	0 (0%)	1 (1.67%)			
Cardio	8 (11.43%)	0 (0%)	8 (13.33%)			
Gastroenterology	9 (12.86%)	1 (10%)	8 (13.33%)			
Hematology	1 (1.43%)	1 (10%)	0 (0%)			
Infectious Disease	14 (20.00%)	3 (30%)	11 (18.33%)			
Nephrology	2 (2.86%)	0 (0%)	2 (3.33%)			
Neuro	13 (18.57%)	1 (10%)	12 (20.00%)			
Oncology	1 (1.43%)	0 (0%)	1 (1.67%)			
Ortho/Trauma	8 (11.43%)	1 (10%)	7 (11.67%)			
Pulmonology	12 (17.14%)	3 (30%)	9 (15.00%)			
Vascular	1 (1.43%)	0 (0%)	1 (1.67%)			
Disposition	0.1037					
Acute Rehabilitation	10 (14.29%)	0 (0%)	10 (16.67%)			
Home	18 (25.71%)	0 (0%)	18 (30.00%)			
Home w/ Home Health	5 (7.14%)	2 (20%)	3 (5.00%)			
Hospice Medical Facility	2 (2.86%)	0 (0%)	2 (3.33%)			
LTACH ^g^	6 (8.57%)	1 (10%)	5 (8.33%)			
Morgue	18 (25.71%)	5 (50%)	13 (21.67%)			
SNF ^h^	11 (15.71%)	2 (20%)	9 (15.00%)			

a Chronic obstructive pulmonary disease.

b Carbapenem-Resistant Organism.

c Substance use disorder / Intravenous drug use.

d Activities of daily living.

e Only includes those tested at UKHC; all outside tests (n = 12) excluded for this variable.

f Intensive care unit.

g Long-Term Acute Care Hospital.

h Skilled Nursing Facility.

I Multivariable logistic regression.

## Discussion

In February 2023, UKHC implemented a targeted *C. auris* screening program for inpatients identified as high-risk based on internally defined criteria. The number of monthly tests ranged from 480 to 1,210, with a positivity rate of 0.96% on average. The proportion of community-onset cases increased from 8% to 54% with the implementation of *C. auris* screening. In univariable analyses, diabetes mellitus, osteomyelitis, tube feeding, tracheostomy, and urinary diversion were significantly associated with clinical infection compared to colonization. However, none of these variables remained statistically significant in multivariable logistic regression. Among colonized patients, the primary reasons for testing were ICU admission, point prevalence, and wound-related concerns.

Targeted screening facilitated early identification of colonized patients, enabling timely implementation of contact precautions and potentially preventing *C. auris* transmission to other hospitalized patients. Implementing targeted inpatient screening was feasible, with a relatively low positivity rate (∼1%). However, the absolute number of detected cases was 3.56 per month, demonstrating the utility of this strategy in identifying colonized patients and mitigating exposure risk. Similar rates have been reported in studies with comparable testing criteria.^6–8^ Following implementation, the proportion of community-onset cases increased from 8% to 54%, likely reflecting improved detection of colonized patients. While no outbreaks were observed before implementation, two occurred afterward, involving three and seven associated patients, respectively. Despite enhanced screening efforts, these outbreaks highlight the persistent risk of *C. auris* transmission, emphasizing the need for stringent infection control measures and continued surveillance.

Currently, there is no official guidance on screening opportunities from major public health entities; however, medical centers have implemented programs based on known risk factors.^2,4–10^ Risk factors include being critically ill or immunocompromised, elderly age, diabetes mellitus, recent surgery, the presence of indwelling medical devices, the use of hemodialysis, a neutropenic state, chronic renal disease, mechanical ventilation, or the use of broad-spectrum antibiotic and/or antifungal drugs.^1,3,5,7,10^ Screening protocols that have been developed comprise admission from other countries (either direct admission or 24 h duration) or the presence of an MDRO,^4^ admission from long-term acute care hospitals, outside hospitals, and skilled nursing facilities, and ICU admissions.^5–8^

Of the 71 positive cases, 10 were clinical infections. Among these, five underwent screening within ±30 days of a positive clinical specimen, with four testing positive, yielding an estimated sensitivity of 80%. This finding suggests that a negative screening result may not reliably exclude the presence of *C. auris*, particularly in patients with intermittent or low-level colonization. Additionally, 2 of 7 patients with previously known colonization tested negative upon repeat *C. auris* screening, further underscoring the challenges of detecting colonization. Variability in colonization burden, sample collection techniques, or fluctuations in microbial load may contribute to these discrepancies.

This study has several limitations. The small sample size may have limited the ability to detect additional associations between clinical infection and colonization, along with generating concern about statistical power. Additionally, the study population reflects UKHC’s patient demographics and may not be generalizable to other U.S. healthcare settings. The cost per *C*. *auris* PCR test is approximately $50, which presents a potential financial burden. Although cost analysis was not within the scope of this study, a formal cost–benefit evaluation may be warranted in future work. Lastly, no demographic or clinical data were collected for negative patients, limiting comparative assessments.

In conclusion, the implementation of a targeted *C. auris* screening program at UKHC provided valuable insights into epidemiologic trends, patient demographics, and potential risk factors. Understanding these factors is critical for optimizing infection prevention strategies, refining screening protocols, and informing public health initiatives to mitigate *C. auris* transmission in healthcare settings.

## Supporting information

10.1017/ash.2025.10151.sm001 Fursman et al. supplementary material 1 Fursman et al. supplementary material

10.1017/ash.2025.10151.sm002 Fursman et al. supplementary material 2 Fursman et al. supplementary material

## Supplementary material

The supplementary material for this article can be found at https://doi.org/10.1017/ash.2025.10151.
