# Supplementary material for: Candida auris screening, positivity trends, and patient characteristics at the University of Kentucky between 2021 and 2024
Source: Antimicrob Steward Healthc Epidemiol. 2025 Oct 14;5(1):e258. doi: 10.1017/ash.2025.10151 (PMC12538337; doi:10.1017/ash.2025.10151)
Supplement: Fursman et al. supplementary material 1 — Fursman et al. supplementary material [file S2732494X25101514sup001.docx]

**Supplemental Table 1. Distribution of Pre- and Post-Implementation Classification for *Candida auris* Positive Patients**

|  | Pre-implementation (n=13) | Post-implementation (n=57) |
| --- | --- | --- |
| Onset |  |  |
| Community | 1 (7.69%) | 27 (57.37%) |
| Hospital | 7 (53.85%) | 26 (45.61%) |
| Outside Facility | 5 (38.46%) | 4 (7.02%) |
| Specimen Type |  |  |
| Clinical | 6 (46.15%) | 4 (7.02%) |
| Colonization | 7 (53.85%) | 53 (92.98%) |
| Point Prevalence Testing | 2 (15.38%) | 10 (17.54%) |

**Supplemental Table 2. *Candida auris* Clinical Case Specific Information**

|  | Clinical Cases (n=10) |
| --- | --- |
| Specimen type |  |
| BAL | 5 |
| Urine | 5 |
| CSF | 1 |
| Blood | 2 |
| Wound | 2 |
| Treatment |  |
| No treatment | 3 |
| Micafungin 100mg | 4 |
| Micafungin 150mg | 3 |
| Surveillance Screening |  |
| Surveillance testing present ±30 days from clinical specimen | 5 |
| All surveillance testing negative | 1 |

**Supplemental Figure 1. Positive *Candida auris* Case Distribution**
