# Supplementary figures and images for: Candida auris screening, positivity trends, and patient characteristics at the University of Kentucky between 2021 and 2024
Source: Antimicrob Steward Healthc Epidemiol. 2025 Oct 14;5(1):e258. doi: 10.1017/ash.2025.10151 (PMC12538337; doi:10.1017/ash.2025.10151)

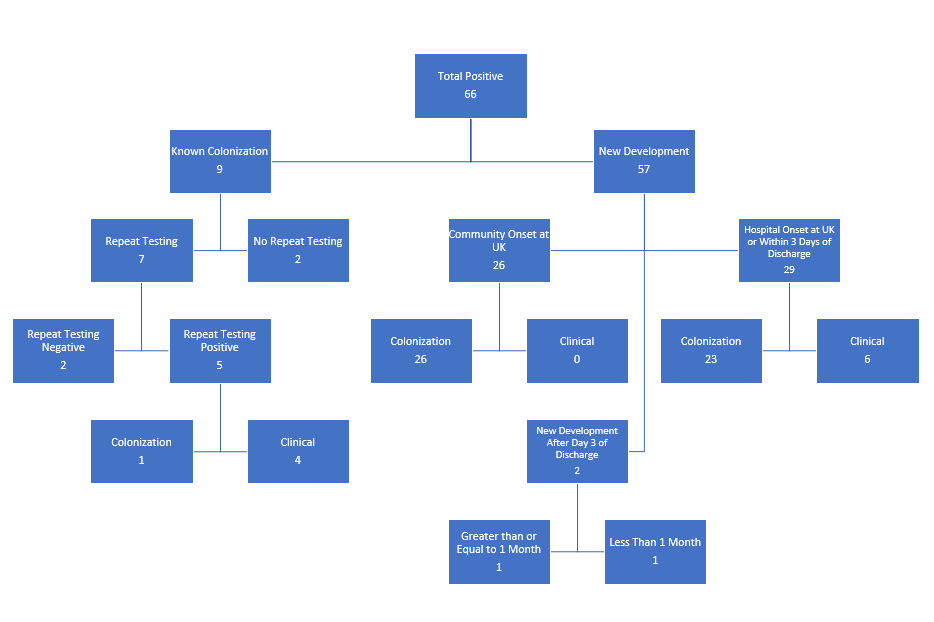

Supplement: Fursman et al. supplementary material 2 — Fursman et al. supplementary material [file S2732494X25101514sup002.tiff]
